# Effect of goal-directed fluid therapy based on plasma colloid osmotic pressure on the postoperative pulmonary complications of older patients undergoing major abdominal surgery

**DOI:** 10.1186/s12957-023-02955-5

**Published:** 2023-02-28

**Authors:** Anqi Feng, Pan Lu, Yanan Yang, Ying Liu, Lei Ma, Jianrui Lv

**Affiliations:** Department of Anesthesiology, Second Affiliated Hospital of Xi’an Jiaotong University, Xi’an, 710004 Shanxi China

**Keywords:** Goal-directed therapy (GDT), Plasma colloid osmotic pressure (COP), Stroke volume variation (SVV), Postoperative pulmonary complication

## Abstract

**Background:**

As an important component of accelerated rehabilitation surgery, goal-directed fluid therapy (GDT) is one of the optimized fluid therapy strategies and is closely related to perioperative complications and mortality. This article aimed to study the effect of combining plasma colloid osmotic pressure (COP) with stroke volume variation (SVV) as a target for intraoperative GDT for postoperative pulmonary complications in older patients undergoing major abdominal surgery.

**Methods:**

In this study, older patients (*n* = 100) undergoing radical resection of gastroenteric tumors were randomized to three groups: Group C (*n*1 = 31) received a conventional infusion regimen, Group S1 (*n*2 = 34) received GDT based on SVV, and Group S2 (*n*3 = 35) received GDT based on SVV and COP. The results were recorded, including the lung injury score (LIS); PaO_2_/FiO_2_ ratio; lactic acid value at the times of beginning (T0) and 1 h (T1), 2 h (T2), and 3 h (T3) after liquid infusion in the operation room; the total liquid infusion volume; infusion volumes of crystalline and colloidal liquids; urine production rate; pulmonary complications 7 days after surgery; and the severity grading of postoperative pulmonary complications.

**Results:**

The patients in the S2 group had fewer postoperative pulmonary complications than those in the C group (*P* < 0.05) and the proportion of pulmonary complications of grade 1 and higher than grade 2 in S2 group was significantly lower than that in C group (*P* <0.05); the patients in the S2 group had a higher PaO_2_/FiO_2_ ratio than those in the C group (*P* < 0.05), lower LIS than those in the S1 and C groups (*P* < 0.05), less total liquid infusion than those in the C group (*P* < 0.05), and more colloidal fluid infusion than those in the S1 and C groups (*P* < 0.05).

**Conclusion:**

The findings of our study show that intraoperative GDT based on COP and SVV can reduce the incidence of pulmonary complications and conducive to shortening the hospital stay in older patients after gastrointestinal surgery.

**Trial registration:**

Chinese Clinical Trial. no. ChiCTR2100045671. Registry at www.chictr.org.cn on April 20, 2021.

## Introduction

Gastrointestinal surgery is a high-risk operation [1, 2]. Although the surgical operation and perioperative treatment have been greatly improved, the incidence of postoperative complications and surgical mortality are still high, especially in older patients. Older patients tend to have severe internal disorders and low system function before surgery, which could lead to a higher risk of major complications, such as pulmonary complications, seriously affecting the postoperative rapid recovery of patients and prolonging the length of hospital stay [3].

Enhanced recovery after surgery (ERAS) protocols are increasingly widely used in the perioperative treatment and can greatly improve patient outcomes, shorten postoperative hospital stays, reduce perioperative complications, and decrease readmission rates to the hospital by 30–50% [4]. Perioperative fluid management plays a pivotal role in the implementation of ERAS protocols.

Numerous observational studies have reported a strong association between both hypovolemia and overloaded intraoperative fluid infusion and an increased risk of postoperative complications [5–7]. Previous studies have shown a strong correlation between appropriate perioperative fluid management and a reduction in the incidence of postoperative pulmonary complications in patients who undergo major gastrointestinal surgery [8, 9].

Adequate fluid intake to maintain cardiac output and blood pressure can ensure tissue perfusion, but it does not indicate a good state of microcirculation and tissue oxygenation. Therefore, static indicators cannot accurately guide fluid perfusion in the perioperative period. With the development of science and technology, dynamic indicators have been widely used in perioperative fluid management, such as stroke volume variation (SVV), and many good effects have been achieved [10]. Previous studies have shown that SVV can predict liquid reactions to some extent.

It is important to monitor not only systemic responses to fluid therapy but also microcirculation and homeostasis. However, research focusing on patients’ terminal tissue perfusion and oxygenation when receiving perioperative fluid management based on more advanced dynamic indicators is lacking. Plasma colloid osmotic pressure (COP) is an important factor in maintaining the balance of fluid flow between the extravascular and intravascular lumens. COP can inhibit the movement of water from intravascular to extravascular and allows interstitial fluid to infiltrate back into the blood vessels from the postcapillary venule, which plays an important role in stabilizing blood volume and preventing tissue edema. A report on pulmonary edema seems to indicate that the maintenance of a normal COP may be of greater importance in critically ill patients [11]. In our study, we hypothesized that the intraoperative use of COP measurements might be valuable for fluid intake balance and be significant for reducing postoperative pulmonary complications.

## Materials and methods

### Study design

#### Ethics

The protocol of the current study was approved by the department of anesthesiology in the Second Affiliated Hospital of Xi’an Jiaotong University. Approval for this study was provided by the medical ethics committee (China). Written informed consent was obtained from all enrolled patients. The trial was registered with the medical ethics committee of the Second Affiliated Hospital of Xi’an Jiaotong University (the code of ethics: 2,020,138). This trial was registered by Anqi Feng with the Chinese Clinical Trial Registry on April 20, 2021 (number ChiCTR2100045671). Patients were screened for eligibility from December 2020 to December 2021. A total of 180 patients scheduled for elective major abdominal surgery were enrolled in this prospective study.

#### Sample size estimation

The primary outcomes of our previous work show that the incidence of postoperative pulmonary complications was 30% in Group C, 5% in Group S1, and 5% in Group S2. The ratio of the experimental group and control group was 1:1. Unilateral test was adopted, with *α* error value of 0.05 and *β* error value of 0.2, confidence interval of 95%. A single sample size of 26 people was needed to calculate the incidence of Groups C and S1. A single sample size of 26 people was needed to calculate the incidence of Groups C and S2. Then, the total sample size was 78 (the formula is shown as follows). In the process of previous work, due to the influence of COVID-19, the lost follow-up rate was 55%. Thus, we set the anticipated dropout rate as 55% when the sample size was calculated, and the total sample size required is 174 (58 in Group C; 58 in Group S1; 58 in Group C). Then, to be conservative, we decided to settle the sample size at 180. A total of 180 patients scheduled for elective major abdominal surgery were enrolled in this prospective study.

$$n=({z}_{1-\frac{\alpha }{1}}+{z}_{1-\beta }{)}^{2}(s{d}_{1}^{2}+s{d}_{2}^{2})/(mea{n}_{1}-mea{n}_{2}{)}^{2}$$

#### Inclusion criteria and exclusion criteria

The inclusion criteria were adult patients ≥ 65 years old undergoing elective open radical resection of gastroenteric tumors (radical gastrectomy includes total gastrectomy, partial gastrectomy at the upper root, and partial gastrectomy at the lower root; radical resection of rectal cancer includes Dixon surgery, Miles surgery, Hartmann surgery; radical colon cancer operations include radical right colon cancer, radical left colon cancer, transverse colon cancer, and sigmoid colon cancer), body mass index (BMI) 15–30 kg m^−2^, and ASA grades I or II. Exclusion criteria were patients with hypertension and poor blood pressure control (blood pressure above 160/90 mmHg or irregular medication), diabetes, intraoperative blood loss > 500 ml or intraoperative infusion of blood products, hypersensitivity to hydroxyethyl starch, impaired liver and kidney function (transaminase more than 2 times the normal value, creatinine more than 1.5 times the normal value), operation time < 60 min, patients with severe cardiovascular disease (NYHA grade III or above), preoperative plasma COP seriously deviated from the normal range, recent (≤ 2 weeks) history of severe pulmonary dysfunction, respiratory failure, emergency surgery, neuromuscular disease, and severe pulmonary bulla.

### Study procedures and measures

#### Randomization

Eligible patients were randomized into the traditional infusion group (Group C), GDT based on the SVV group (Group S1), or GDT based on SVV and COP groups (Group S2). The subjects did not know which group they were assigned to and were randomized using Research Randomizer (http://www.randomizer.org/) by a research assistant who did not know the experimental scheme.

#### Intraoperative monitoring

The surgical procedure was performed under general anesthesia. The ECG, heart rate, arterial blood pressure, SpO_2_, urine volume, end-tidal carbon dioxide pressure (ETCO_2_), and depth of anesthesia were measured continuously upon entering the operation room for all patients. According to the results of blood gas analysis and gas monitoring, the respiratory rate or tidal volume was adjusted to maintain ETCO_2_ at 35 ~ 45 mmHg. Intraoperative heat preservation treatment was performed as follows: the body temperature was maintained at 36 ~ 37 ℃. If the patient’s blood pressure was 30% lower than the blood pressure base value or systolic blood pressure was lower than 80 mmHg, an intravenous pump with a small dose of norepinephrine (0.2 ~ 0.4 µg kg^−1^ h^−1^) was used. Nicardipine was intravenously injected at 10 µg kg^−1^ to maintain intraoperative blood pressure within the normal range if blood pressure was 30% higher than the base value or systolic pressure was higher than 160 mmHg. When the patient’s heart rate was lower than 50 beats h^−1^, atropine was intravenously injected at 0.5 µg kg^−1^, and when the heart rate was higher than 100 beats h^−1^, 0.5 mg kg^−1^ esmolol was slowly injected intravenously. If the intraoperative COP was < 20 mmHg, furosemide 5 mg was intravenously injected.

#### Infusion treatment

All patients were randomly divided into 3 groups. In Group S1, patients received a continuous infusion of crystal solution (Ringer’s acetate solution) and colloid solution (hydroxyethyl starch 130/0.4 sodium chloride injection) through two devices connected to the peripheral vein. Vigileo was used to measure the SVV and control the fluid infusion rate to keep the SVV below 13%. The patients in Group S2 were monitored with the same parameters as those used in Group S1, the intraoperative COP of patients in Group S2 was monitored simultaneously, and the volume and proportion of the infusion of crystal and colloid liquid were adjusted by controlling the relative infusion speed of the two fluids so that the plasma colloid osmotic pressure of patients was maintained at 20 ~ 25 mmHg. We collected the upper serum of the blood sample after centrifugation and measured COP with a colloid osmotic pressure monitor (ONKOMETER BMT923). Patients in Group C received a conventional infusion regimen, and the total amount of input fluid = physiological requirements + preoperative cumulative loss + continued loss + third gap loss + compensatory volume expansion. Compensatory expansion capacity was supplemented with compound Ringer’s solution at 7 ml kg^−1^, physiological requirements and preoperative cumulative loss were supplemented with acetic acid Ringer’s solution according to the 4–2-1 rule, the continued loss was supplemented with hydroxyethyl starch 130/0.4 sodium chloride injection in equal amounts, and third gap loss was supplemented with compound Ringer’s solution at 5 ml kg^−1^ h^−1^). Compensatory volume expansion was supplemented before anesthesia, and the remaining fluid volume was infused after induction of anesthesia and during surgery.

#### Primary outcomes

The primary outcome was that the incidence of postoperative pulmonary complications, including pneumonia or bronchitis, pulmonary edema, atelectasis, and pleural effusion were recorded within 7 days after surgery. And the Clavien-Dindo classification was used to grade the severity of postoperative pulmonary complications [12]. In Grade 0, there are no signs of pulmonary complications. Grade 1 included minor risk events not requiring therapy, and the chest radiograph was normal. Grade 2 included moderate to severe cough, bronchospasm, atelectasis, and requiring pharmacological treatment with drugs (pneumonia treated with antibiotics on the ward). Grade 3 postoperative presence or combination of pleural effusion requiring pleural puncture, confirmed pneumonia, pneumothorax, postoperative intubation, and ventilator dependence time ≤ 48 h. Grade 4 postoperative respiratory failure and multiorgan dysfunction (lung failure requiring intubation). Grade 5 is the death of a patient.

#### Secondary outcomes

We assessed the following secondary outcomes: perioperative injury score (LIS), partial pressure of inspiration of oxygen (PaO_2_/FiO_2_) ratio, lactate levels, total infusion volume, infusion volume of crystalline liquid, infusion volume of colloidal liquid, and urine volume. Intraoperative and postoperative data were collected by researchers who were blinded to the study group assignments.

#### Lung injury score (LIS) and pulmonary function

LIS was measured by a pulmonary ultrasound, which was examined by a Sonosite portable color ultrasound machine according to the pulmonary BLUE protocol to monitor pulmonary extravascular fluid content and predict the degree of lung injury. Oxygenation was quantified by calculating the PaO_2_/FiO_2_ ratio (arterial partial pressure of oxygen PaO_2_/inspiratory oxygen concentration FiO_2_) for each patient at each point of measurement as long as the patient was intubated and mechanically ventilated.

All the parameters of secondary outcomes were measured at the time of beginning infusion (T0) and 1 h (T1), 2 h (T2), and 3 h (T3) after infusion in the operating room.

### Statistical analysis

SPSS 22.0 software was used for data processing. Shapiro–Wilk test was used to test the normality of the data, a *P* value > 0.05 was considered as a normal distribution. Normally distributed measurement data are expressed as the mean ± standard deviation, the two-factor repeated measure ANOVA (mixed ANOVA) was used for comparisons among the three groups and each time point. Nonnormally distributed measurement data are expressed as the median (inter-quartile range). Kruskal–Wallis test was used for comparisons among the three groups. Counting data were expressed as the proportion, and chi-square test was used. A *P* value < 0.05 was considered to be significant.

## Results

### Participant flow

**Fig. 1:**
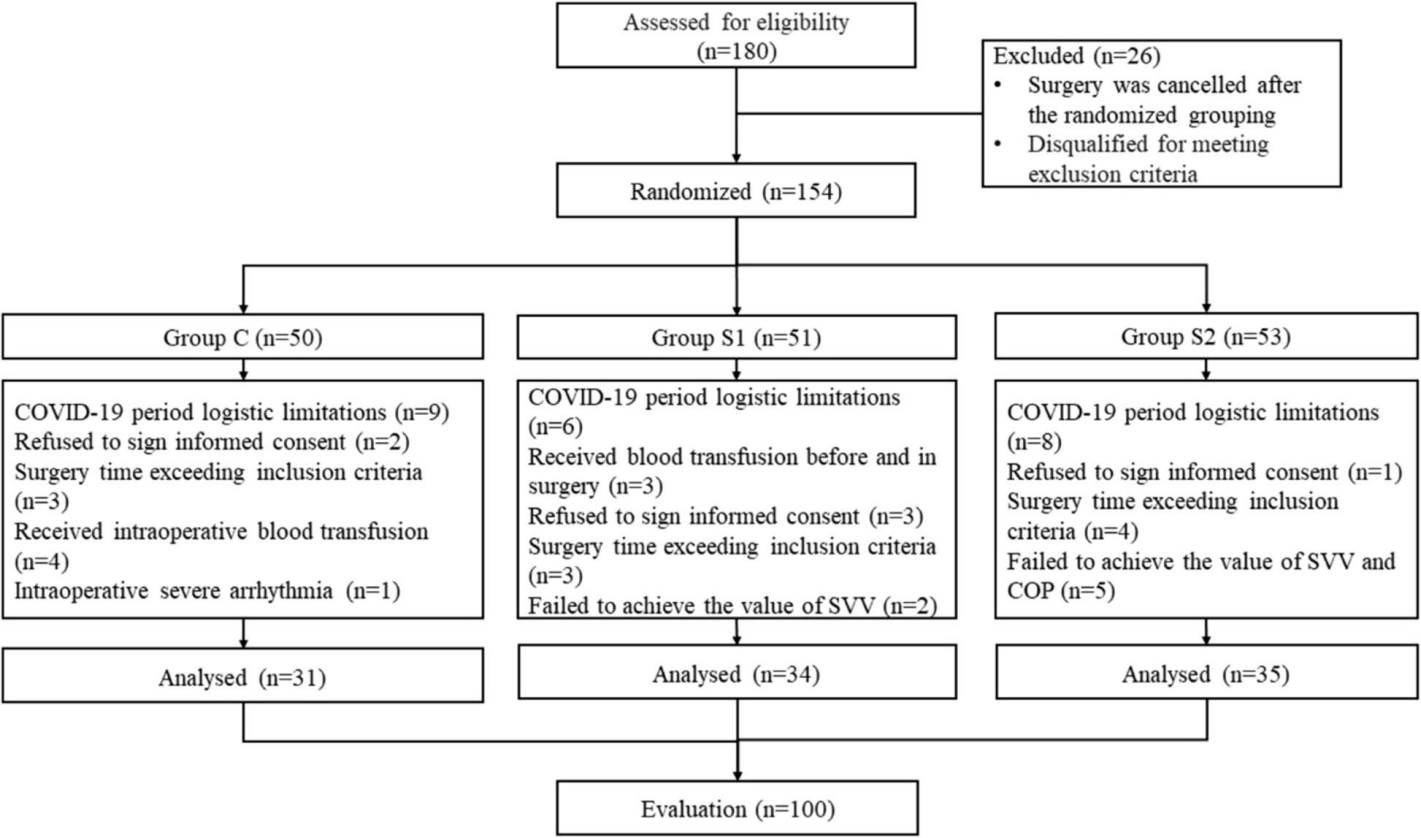
Flow diagram of enrolled patients

A total of 180 patients were randomized after recruitment from the gastrointestinal surgery department of our hospital. Among those subjects, 80 patients (29 patients in the C group, 26 patients in the S1 group, 25 patients in the S2 group) were excluded from the analysis. Eight subjects in the C group, 4 subjects in the S1 group, 2 subjects in the S2 group were excluded because their surgery was canceled after the randomized grouping, and 2 subjects in the C group, 5 subjects in the S1 group, and 5 subjects in the S2 group were disqualified for meeting exclusion criteria. In the C group, 9 patients failed to undergo follow-up due to logistic limitations during the COVID-19 period, 2 patients refused to sign informed consent for the study, 3 patients failed to undergo follow-up due to surgery time exceeding the inclusion criteria, 4 patients received intraoperative blood transfusions for blood loss greater than 500 ml, and 1 patient had an intraoperative severe arrhythmia. In the S1 group, 6 patients failed to undergo follow-up due to logistic limitations during the COVID-19 period, 3 patients refused to sign informed consent for the study, 3 patients failed to undergo follow-up due to surgery time exceeding the inclusion criteria, 2 patients received intraoperative blood transfusion for blood loss greater than 500 ml, 1 patient received a blood transfusion due to anemia before surgery, and 2 patients failed to achieve the value of preoperative SVV. In the S2 group, 8 patients failed to undergo follow-up due to logistic limitations during the COVID-19 period, 1 patient refused to sign informed consent for the study, 4 patients failed to undergo follow-up due to surgery time exceeding the inclusion criteria, and 5 patients failed to achieve the value of preoperative SVV and COP. Thus, 31 patients in the C group, 34 patients in the S1 group, and 35 patients in the S2 group were included in the final analysis as shown in Fig. 1. For the gap between the patients initially included and those included in the final assessment, we performed analyses between two subgroups and the results showed a low probability of selection bias (Table 1).

**Table 1:** Characteristics for predicted and actual samples

Parameters	Expected sample (*n* = 180)	Actual sample (*n* = 100)	*P* value
Age (year)	64.3 ± 2.7	63.9 ± 3.3	0.38
Gender (male/female)	88/92	51/49	0.27
BMI (kg m^−2^)	23.9 ± 4.7	24.3 ± 4.5	0.17
Duration of surgery (min)	181.7 ± 60.5	178.2 ± 59.7	0.49
Duration of anesthesia (min)	210.4 ± 62.3	219.1 ± 71.1	0.39
Stomach/intestinal surgery	91/89	51/49	0.12
Smoker	67 (37)	35 (35)	0.25

### Baseline parameters and operative characteristics

Patient characteristics and comorbidities are shown in Table 2. Patients did not differ significantly in age, sex, BMI (body mass index), duration of surgery, duration of anesthesia, the type of operation, preoperative respiratory function, or smoking history (*P* > 0.05).

**Table 2:** General characteristics of patients

Parameters	Group C (*n*1 = 34)	Group S1 (*n*2 = 31)	Group S2 (*n*3 = 35)	*P* value
Age (year)	63.3 ± 3.6	64.4 ± 3.6	64.2 ± 2.7	0.24
Gender (male/female)	19/15	14/17	18/17	0.65
BMI (kg m^−2^)	23.9 ± 4.7	24.3 ± 3.3	24.8 ± 5.1	0.37
Duration of surgery (min)	172.8 ± 59.5	179.3 ± 61.2	182.5 ± 58.4	0.54
Duration of anesthesia (min)	219.6 ± 79.2	220.9 ± 60.6	217 ± 71.4	0.97
Stomach/intestinal surgery	16/18	15/16	20/15	0.76
Preoperative respiratory function				
Normal	24 (70)	23 (74)	23 (66)	0.19
Mild ventilation dysfunction	6 (18)	5 (16)	7 (20)	0.48
Moderate ventilation dysfunction	4 (12)	3 (8)	5 (15)	0.25
Smoker	12 (35)	10 (32)	13 (37)	0.53

The perioperative SVV was controlled below 13% in Groups S1 and S2, and the COP was controlled above 20 mmHg in Group S2. The value of SVV at T1, T2, and T3 was significantly lower than the value at T0 in Groups S1 and S2 (*P* < 0.05), and the value of COP at T1, T2, and T3 was significantly lower than the value at T0 in Group S2 (*P* < 0.05). There was no difference in SVV between Groups S1 and S2 (*P* > 0.05) (Table 3).

**Table 3:** SVV value in the Group S1 and COP and SVV values in the Group S2 (‾*x* ± *s*)

Group	Parameters	T0	T1	T2	T3
S1	SVV (%)	12.9 ± 2.8	8.6 ± 2.2^*^	8.6 ± 1.3^*^	8.8 ± 1.7^*^
S2	SVV (%)	12.3 ± 2.8	8.5 ± 1.4^*^	8.9 ± 1.9^*^	8.7 ± 2.0^*^
	COP (mmHg)	19.5 ± 2.7	22.3 ± 1.5^*^	22.1 ± 2.2^*^	21.9 ± 1.6^*^

Data are expressed as mean ± standard deviation

Compared with T0 in the same group, ^*^P < 0.05 is statistically significant

### Primary outcomes

#### Incidence and classification of major complications

The total postoperative pulmonary complications and postoperative pulmonary edema in Group S2 were significantly lower than those in Group C (*P* < 0.05). The proportion of pulmonary complications of grade 1 and higher than grade 2 in Group S2 was significantly lower than that in Group C (*P* < 0.05). There was no significant difference between Group S1 and Group C (*P* > 0.05). The data are presented in Table 4.

**Table 4:** Incidence and classification of postoperative pulmonary complications [number of cases (%)]

Parameters	Group C (*n*1 = 34)	Group S1 (*n*2 = 31)	Group S2 (*n*3 = 35)
Incidence of postoperative pulmonary complications			
Pleural effusion	0 (0)	1 (3)	0 (0)
Pneumonia or bronchitis	2 (5)	1 (3)	1 (3)
Pulmonary edema	4 (12)	2 (6)	0 (0)^#^
Total number of postoperative pulmonary complications	6 (19)	4 (12)	1 (3)^#^
Classification of postoperative pulmonary complications			
Grade 0	28 (81)	27 (88)	34 (97)
Grade 1	4 (12)	3 (10)	1 (3)^#^
≥ Grade 2	2 (5)	1 (3)	0 (0)^#^

Data are expressed as a number of cases (percentage of total)

Compared with group C, ^#^*P* < 0.05 is statistically significant

### Secondary outcomes

#### Lung injury score (LIS)

Overall, the postoperative lung injury scores were significantly higher than the preoperative scores (*P* < 0.05). The proportion of this upwards trend was more obvious in Group C, and although the elevations appeared to be more numerous in S1 than S2, there was no significant difference in statistical analysis (*P* > 0.05). The data are presented in Table 5.

**Table 5:** Lung injury score (LIS) [median (inter-quartile range)]

Group	T0	T1	T2	T3
C	0 (0)	0 (0)	1.5 (1)^*#^	1 (1)^*#^
S1	0 (0)	0 (0)	1 (1)^*^	1 (1)^*#^
S2	0 (0)	0 (0)	1 (1)^*^	0 (1)

Data are expressed as median (inter-quartile range)

Compared with T0 in the same group, ^*^P < 0.05 is statistically significant

Compared with group S2 at the same time, ^#^*P* < 0.05 is statistically significant

#### ***PaO***_***2***_***/FiO***_***2***_*** ratio***

**Fig. 2:**
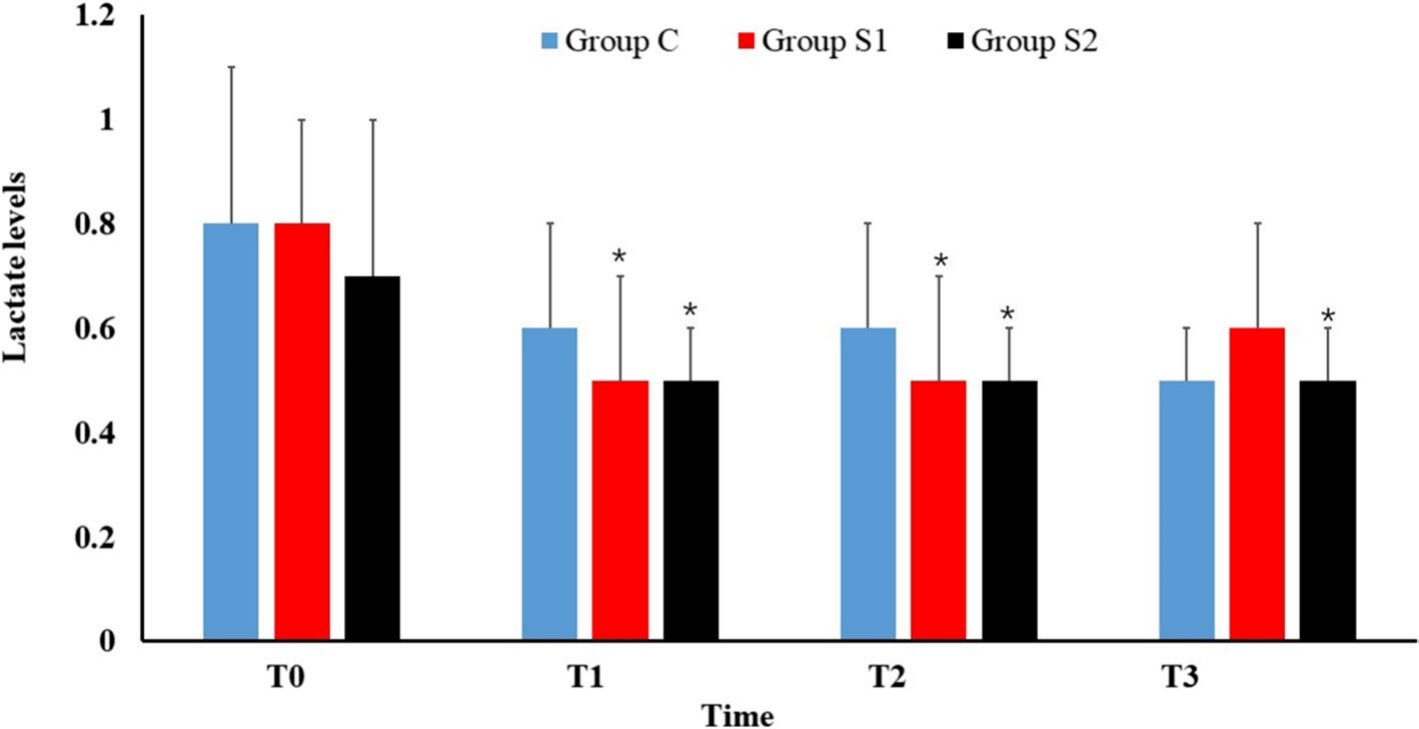
PaO_2_/FiO_2_ ratio. Data are expressed as mean ± standard deviation. Compared with T0 in the same group, ^*^P < 0.05 is statistically significant. Compared with group C at the same time, ^#^*P* < 0.05 is statistically significant

The PaO_2_*/*FiO_2_ ratio of patients in Group S2 was significantly higher than that in Group C at T2 and T3 (*P* < 0.05). In Group S2, the PaO_2_*/*FiO_2_ ratio decreased slightly from 699.6 ± 61.2 mmHg to 652.3 ± 45.5 mmHg. The PaO_2_*/*FiO_2_ ratio of patients at T3 was significantly lower than the ratio at T0 in Group S1. In Group S1 (*P* < 0.05), a slow decrease in the PaO_2_*/*FiO_2_ ratio was observed after 2 h (mmHg). There was no significant difference in the PaO_2_*/*FiO_2_ ratio between Group C and Group S1 at the same time point (*P* > 0.05). The lowest mean PaO_2_*/*FiO_2_ ratio was observed in Group C at T3 (589.5 ± 36.9 mmHg), and its decline was the greatest. The PaO_2_*/*FiO_2_ ratio of patients at T2 and T3 was significantly lower than the ratio at T0 in Group C (*P* < 0.05). The data are presented in Fig. 2.

#### Metabolic data

**Fig. 3:**
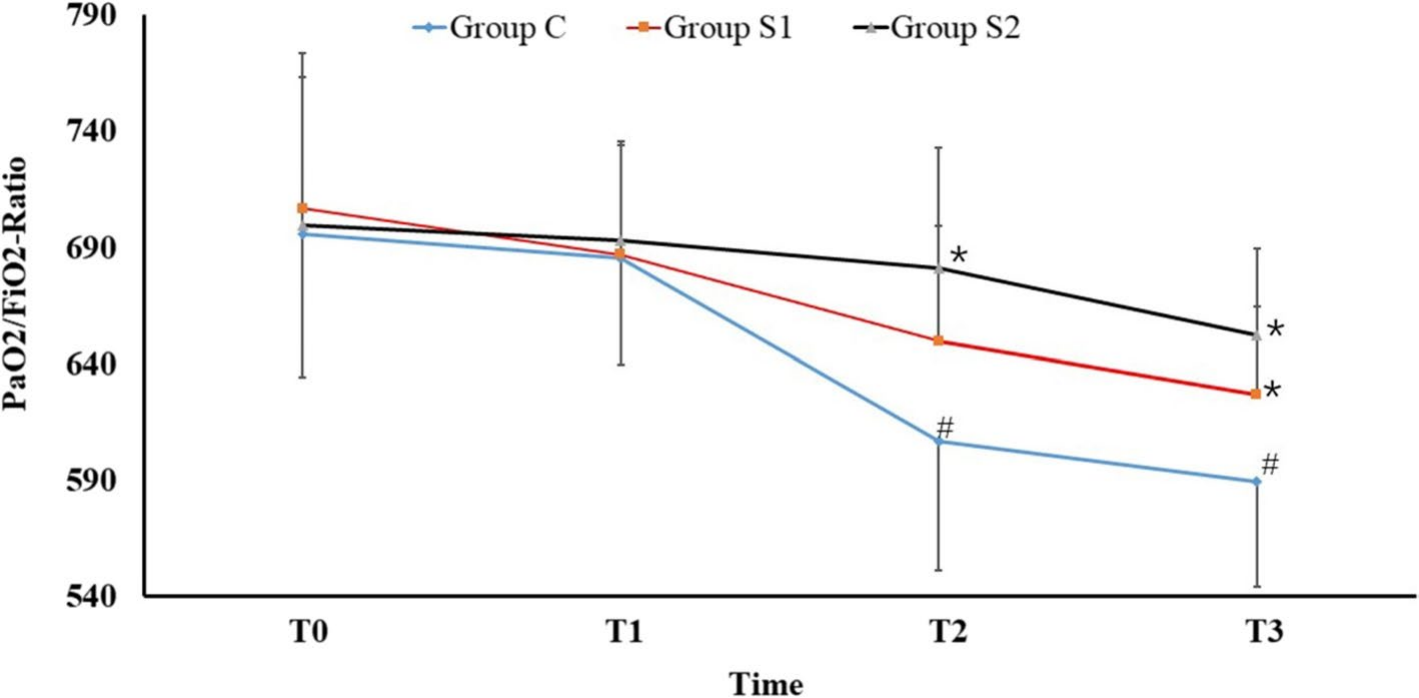
Lactate levels. Data are expressed as mean ± standard deviation. Compared with T0 in the same group, *P < 0.05 is statistically significant

Arterial blood gas analysis mainly refers to lactate levels and is given in Fig. 3. Lactate levels decreased significantly at T2, T3, and T4 compared to lactate levels at T0 in Group S2 (*P* < 0.05). A significant decrease was also observed at T1 and T2 compared with T0 in Group S1 (*P* < 0.05). There was no significant difference in lactate levels among the four times in Group C (*P* > 0.05). There was no significant difference among the three groups (*P* > 0.05). At this point, it has to be clearly stated that all significant changes in all metabolic data were well within normal values and are considered clinically irrelevant.

#### Hydrodynamic analysis

Data regarding fluid administration and urine production are presented in Table 6. Compared with Group S2, Groups C and S1 had more fluid infusion (*P* < 0.01) and a higher urine production rate (*P* < 0.05). At the observed time point, the amount of colloid liquid infusion in Group S2 was higher than that in the other two groups, and the time point of colloid liquid infusion was earlier than that in the other two groups (*P* < 0.05). There was no significant difference between Group S1 and Group C (*P* > 0.05).

**Table 6:** Hydrodynamic analysis (‾*x* ± *s*)

Group	Total infusion volume (ml)	Infusion volume of crystalline liquid (ml)	Infusion volume of colloidal liquid (ml)	Urine production rate(ml min^−1^)
C	3200 ± 303	2500 ± 500	220 ± 78^†^	5.45 ± 0.87^†^
S1	2241 ± 272^#^	1983 ± 269^#^	266 ± 88^†^	5.08 ± 1.13^†^
S2	2258 ± 304^#^	1844 ± 575^#^	450 ± 83	3.72 ± 0.40

Data are expressed as mean ± standard deviation

Compared with group C, ^#^*P* < 0.05 is statistically significant

Compared with group S2, ^†^*P* < 0.05 is statistically significant

## Discussion

This study shows that COP- and SVV-based GDT protocols can more accurately improve intraoperative pulmonary edema, lowering the incidence of postoperative pulmonary complications of major abdominal surgery. Importantly, intraoperative COP- and SVV-based GDT reduced postoperative pulmonary complications of grade 2 and higher severity, which contributed to improving the outcome of major abdominal surgery, and conducive to shortening postoperative hospital stay (Table 4).

Some studies have shown that perioperative GDT seems to be more beneficial for patients with higher surgical risk [13, 14]. Many studies have also confirmed the important role of GDTs in major abdominal surgery [15, 16]. It is suggested that GDT guided by SVV can improve the intravascular volume status by controlling the fluid volume as a form of fluid therapy [17, 18], as we found in Group S1 of our study. Compared with the preoperative lactic acid level (T0), the lactic level of the patients in the S1 group decreased to varying degrees after we started GDT guided by SVV (Fig. 3), which indicates that the rapid and targeted intake of sufficient fluid can satisfy tissue perfusion as quickly as possible and reduce blood lactate levels. However, we also found that in the SVV-guided infusion Group S1, lactate levels decreased significantly at T1 and T2 but increased again at T3 (all values were within normal ranges) (Fig. 3). The lactate of patients in the S2 group remained at a lower level. This means that although SVV is a good monitor for fluid infusion in terms of effective circulating blood volume and the response of the cardiovascular system to fluid, it has no good guiding value for providing sensitive monitoring for long-term homeostasis and does not provide good guidance or advice on the choice and use of liquid types.

Therefore, for patients during major abdominal surgery, the perioperative GDT involves not only the maintenance of effective circulating blood volume but also the water balance inside and outside blood vessels. It is important to choose the right type of liquid and reduce the amount of intraoperative fluid infusion and tissue edema by using a reasonable amount of fluid according to the dynamic parameters. According to theoretical analysis, COP is important for maintaining patient fluid balance by influencing fluid flow in and out of blood vessels, according to Starling’s equation. It can more accurately monitor tissue perfusion status.

Studies have shown that increased microvascular permeability in older patients with gastrointestinal diseases leads to extravasation of fluid and protein into the alveoli [2, 19]. COP is mainly provided by serum total protein. This means that the preoperative COP of older patients undergoing gastrointestinal surgery is worse, suggesting that we should pay more attention to the stability of the internal environment. It is suggested that patients with low COP at admission have no significant difference in vital signs, but their hospital stay is significantly longer than those of individuals with normal COP. A previous study showed that COP below 20 mmHg increases interstitial fluid volume and exposes tissues to edema, which in diverse ways may interfere with normal functions [20]. Animal models have shown that existing low COP doubles the fluid leakage from capillaries to the interstitium compared to a similar magnitude increase in hydrostatic pressure. Fluid accumulates in the extracellular space of the lung tissue, forcing the alveoli to collapse and exudate, resulting in alveolar dead space and intrapulmonary shunt, affecting oxygen exchange and increasing the risk of postoperative respiratory failure, pulmonary infection, and acute respiratory distress syndrome [21]. Depressed COP could contribute to pulmonary interstitial fluid overload, which can be assessed by oxygenation indices (PaO_2_*/*FiO_2_ ratio) and chest ultrasound techniques. As we found in our research, patients in the infusion group without COP monitoring presented urorrhagia, an increase in extravascular lung water (EVLW), and a decrease in oxygenation index. As shown in Table 6, the patients in Groups C and S1 had higher urine production rates than those in Group S2. The COP of patients in the S2 group was maintained above 20 mmHg, while the COP of patients in Group C and Group S1 was not monitored. By comparison, the PaO_2_*/*FiO_2_ ratio of patients in Group S2 was significantly higher than that in Group C at T3, and there was no significant decrease compared with that before surgery (Fig. 2). Moreover, among the three groups, only the S2 group had no significant increase in LIS 3 h after surgery (Table 5).

In addition, recent studies have shown that the release of several inflammatory mediators caused by gastrointestinal disease and its correlative excessive COP lead to the degradation of endothelial glycocalyx [22], which increases endothelial permeability, resulting in pulmonary edema and worsening of gas exchange [23]. Therefore, our research indicated that the maintenance of a normal COP is of greater importance in gastrointestinal surgery.

Furthermore, research shows that GDT based on a combination of dynamic indicators of liquid reactivity and other optimized parameters was more accurate than that based on dynamic indicators alone [24]. Accordingly, as we used in Group S2 of our study, we chose both the COP and the SVV to achieve appropriate fluid loading, which not only controls the total volume of fluid but also maintains the balance of the internal and external volumes of blood vessels. As shown in Fig. 2 of our research results, the PaO_2_*/*FiO_2_ ratio of the S2 group was significantly higher than that of the C group at T2 and T3, and compared with patients in the other two groups, after 3 h of surgery, there was no significant decrease in the PaO_2_*/*FiO_2_ ratio, indicating that GDT guided by this combination had more obvious lung protection at the later stage.

Nevertheless, the type and timing of fluid infusion may be important, as we see in Table 6, since patients in the S2 group received more colloid boluses early during surgery, suggesting an earlier optimization of tissue perfusion, and less fluid accumulated in the extracellular space. This may be associated with the “no absorption rule.” Numerous studies have shown that contrary to what we previously thought, except for the renal cortex and medulla, there is no continuous fluid absorption of downstream microvessels under stable circulation conditions; the effect of interstitial protein on fluid flow is very small; and transcapillary flow is also lower than previously thought [25, 26]. It has been clinically observed that the use of crystalloids and colloids does not improve existing tissue edema, most likely because persistent low COP can lead to the inverse relationship between the interstitial protein concentration gradient near the vessel wall and the driving force, the venous ends of continuous capillaries do not undergo fluid reabsorption, and only a small fraction of the solution filtered into the interstitium is returned to circulation through the lymphatic system [27]. Therefore, dynamic monitoring of COP during perioperative fluid therapy is necessary for patients with chronic low COP before surgery. The normal level of COP should be restored as soon as possible, and the stability of COP should be maintained, which is of great significance for the prevention of postoperative pulmonary edema. In addition, given the significantly lower-than-normal COP at the beginning of surgery and the small differences observed at the end of the surgery, it is difficult to bring the value back to normal or even higher. This is also consistent with our research results, as shown in Table 4. The incidence of postoperative pulmonary edema in the S2 group was significantly lower than that in the C group.

Our study has some limitations. First, COP and SVV were monitored only intraoperatively. GDT should be administered throughout the perioperative period, which may lead to more dramatic improvements in patient recovery. Second, this is a single-center study, and multicenter studies may reduce research errors and make the results more accurate. Third, some factors, such as dietary habits, may affect the accuracy of the results, although we have tried to account for many potential confounders in the experiment.

Future research may focus on the influence of the timing of infusion of different fluid types on the COP to further uncover the qualitative and quantitative influence of fluid types and use timing on microcirculatory perfusion and tissue edema.

## Conclusions

The findings of our study show that intraoperative GDT based on the COP and SVV can reduce the incidence of pulmonary complications and conducive to shortening  the hospital stay after gastrointestinal surgery.

## Data Availability

The data that support the findings of this study are available on request from the corresponding author.

## Abbreviations

GDT: Goal-directed fluid therapy
COP: Plasma colloid osmotic pressure
SVV: Stroke volume variation
LIS: Lung injury score
ERAS: The enhanced recovery after surgery
BMI: Body mass index
ETCO2: End-tidal carbon dioxide pressure
EVLW: Extravascular lung water
